# Modern Day High: The Neurocognitive Impact of Social Media Usage

**DOI:** 10.7759/cureus.87496

**Published:** 2025-07-08

**Authors:** Abhijeet Satani, Kshma Kheskani Satani, Param Barodia, Heth Joshi

**Affiliations:** 1 Department of Neuroscience, Satani Research Centre, Ahmedabad, IND

**Keywords:** brainwaves, cognitive impact, dopamine, eeg study, social media

## Abstract

Background and objective

Extensive use of social media raises concerns regarding its psychological and neurophysiological impact. Although behavioral effects have been the focus of earlier research, there are scarce empirical data addressing the degree to which real-time brain activity alters with social media use. This research aimed to examine the neurocognitive impact of social media usage by assessing brainwave activity via electroencephalography (EEG) to determine specific patterns of neural engagement as well as cognitive/emotional responses.

Methods

EEG recordings were obtained from 100 participants with a 24-channel system based on the 10-20 international standard. Participants were healthy adults aged 18-45 years (mean age: 27.4 years), including 52 females and 48 males. Individuals with a history of neurological or psychiatric disorders were excluded. Data were preprocessed with band-pass and notch filtering, artifact rejection by independent component analysis (ICA), common average referencing, epoching, and downsampling. Participants used social media for 30-minute periods, during which neural activity in five frequency bands (Delta, Theta, Alpha, Beta, Gamma) was recorded and analyzed in terms of user interactions and content type.

Results

Social media use caused marked alterations in brainwave activity. Alpha waves declined during engagement, especially with emotionally charged content, which suggests cognitive load and excitation. Beta and Gamma waves are heightened during active interaction and continue after engagement, which may indicate extended cognitive excitation and emotional engagement. Theta and Delta waves increased slightly during passive surfing or extended use, which could indicate introspection and mental exhaustion. Regional examination identified Beta/Gamma predominance in prefrontal and occipital cortices in decision-making and viewing of visual content, and Beta/Theta in parietal cortex during multitasking between platforms.

Conclusions

Our findings show that social media engages in brain reward pathways akin to those seen in addictive behavior, with extended Beta and Gamma activity having the potential to interfere with emotional regulation and attention. These neurophysiological consequences, especially delayed Alpha recovery and increased Delta activity, could bring to the fore new concerns regarding digital fatigue and mental health. The research indicates the importance of platform design interventions and additional longitudinal investigations.

## Introduction

Social media platforms have become ubiquitous and are deeply integrated into modern life, with over 4.9 billion active users globally spending an average of 2.5 hours daily on these platforms [1]. While extensive research has explored the psychological and behavioural effects of social media, including its associations with anxiety, depression, and distorted self-perception, the underlying neurocognitive mechanisms remain insufficiently explored [2]. How does the brain process the rapid influx of curated content, emotional stimuli, and social feedback inherent to platforms like Instagram, TikTok, and X (formerly Twitter)? What neural signatures distinguish passive scrolling from active engagement, and how do these dynamics contribute to prolonged use or mental fatigue? Advances in electroencephalography (EEG), particularly wearable systems, now enable real-time tracking of neural activity during authentic social media engagement, offering unprecedented insights into the phenomenon [3].

Social media came into existence to fulfill the need for instant communication, global outreach, and information sharing in the digital age [4,5]. It allows people to stay connected, share updates, and access real-time news while also serving as a platform for businesses and content creation [6]. Social media has altered how individuals and organisations connect by giving platforms for amusement, education, and awareness, making it an indispensable component of modern life [7,8].

Dopamine-driven reward systems

Social media platforms use the same variable reward system as gambling and substance addiction to get users hooked [9]. Likes and comments in social media have been found to stimulate the ventral striatum, which is rich in dopamine and is involved in reward anticipation [10]. It was found that TikTok’s algorithm causes dopamine to spike when funny videos are followed by serious political videos. There was more Gamma wave (30-100 Hz) activity during high-reward moments, and Gamma power went up by 62% compared to when participants viewed neutral content [11]. According to research, using infinite scrolling interfaces can weaken inhibitory control in the prefrontal cortex, which results in more Beta waves (12-30 Hz) and makes it harder to stop using the interface [12]. The study of 500 users over six months revealed that those who spent more than two hours a day scrolling had a 35% drop in prefrontal impulse control, as shown by a decrease in Beta wave variability [13]. These results are consistent with prior findings comparing social media’s "pull-to-refresh" feature to slot machines, where intermittent rewards perpetuate attention through sporadic dopamine release [14].

The concept of "dopamine fasting," initially popularized in wellness communities, has garnered empirical backing [15]. A 30-day abstinence trial involving 200 participants showed Alpha wave (8-12 Hz) suppression during withdrawal periods (p<0.01), alongside reported symptoms such as cravings and irritability that resemble those seen in substance dependence [16]. Despite these advances, most studies rely on self-reports or fMRI, underscoring a need for real-time EEG data during active social media use, a gap addressed by our study through in-the-moment neural tracking [17].

Advances in EEG methodologies and limitations

Recent innovations in wearable EEG technology have enabled granular analysis of neural responses during real-world social media use [18]. The use of portable 32-channel headsets has been validated through evidence showing sustained Beta activity (15-25 Hz) during Instagram browsing [19]. In a study involving 150 participants, visually stimulating content such as travel photos was found to increase Beta power in the occipital lobe by 28% compared to text-based posts (p<0.05) [20]. In parallel, the Delta/Theta ratio (0.5-8 Hz) has been proposed as a biomarker for cognitive fatigue, with findings indicating that over 30 minutes of social media use significantly elevates Delta wave (0.5-4 Hz) activity in the parietal lobe (p<0.01) [21].

Platform-specific neural effects have also been explored, with comparative analysis revealing 40% higher Gamma activity among TikTok users due to the rapid context-switching characteristic of short-form video feeds [22]. Spectral analysis linked this heightened activity to increased cognitive load in the dorsolateral prefrontal cortex (DLPFC), a brain region involved in task-switching [23]. Additionally, persistent Beta and Gamma activity during newsfeed browsing has been identified as a neural signature of "doomscrolling" [24]. A study involving 300 participants showed delayed Alpha recovery (p<0.001) following exposure to emotionally charged content, such as pandemic updates, which prolonged Beta wave activity by an additional 12 minutes [25].

While wearable EEGs offer improved ecological validity, they are still susceptible to signal noise in uncontrolled environments [26]. Signal processing techniques such as independent component analysis (ICA) have been employed to filter artifacts, though approximately 15% of datasets remain unusable [27]. Furthermore, the majority of existing research emphasizes neural dynamics during use, often overlooking post-engagement effects, a limitation addressed by our extended monitoring of neural activity following social media interaction [28].

Brain region activation

The PFC, critical for executive function, undergoes significant changes during social media use. A 22% reduction in PFC Beta power has been observed after just 20 minutes of engagement, impairing users’ decision-making abilities (p<0.01) [21]. Participants displayed heightened impulsivity, opting for immediate rewards such as ad clicks rather than long-term gains. Further evidence shows that emotionally charged content, particularly outrage-inducing posts, increases coupling between the amygdala and PFC. Combined fMRI-EEG data revealed Gamma surges (35-45 Hz) during such exposure, indicating that emotional stimuli can override rational cognitive control [13].

Social media also activates memory and visual processing regions in the brain. Social comparison behavior has been associated with elevated Theta activity (4-8 Hz) in the temporal lobe, especially when viewing peers’ holiday photos. This 17% Theta increase (p<0.05) reflects the encoding of fear of missing out (FOMO) as a salient memory, potentially reinforcing compulsive checking behavior [22]. In the visual domain, Beta and Gamma dominance in the occipital lobe has been observed during Instagram use, particularly in response to high-contrast imagery such as neon graphics [14]. Building on this, recent data reveal up to a 25% increase in Beta and Theta activity in the parietal lobe during multitasking across platforms (e.g., switching from TikTok to Twitter), signifying a substantial rise in cognitive load compared to single-platform usage (p<0.01) [9].

Mental health and design implications

Prolonged social media use, defined as more than two hours per day, has been associated with increased Delta wave activity (0.5-4 Hz) in adolescents, significantly correlating with self-reported exhaustion (r=0.45, p<0.001) [29]. A longitudinal study involving 1,000 teenagers documented a 50% rise in Delta power over two years among heavy users [30]. Post-pandemic declines in Alpha wave suppression further suggest potential long-term neural adaptation, with adolescents who began heavy use during coronavirus disease 2019 (COVID-19) lockdowns showing an 18% reduction in baseline Alpha power post-pandemic (p<0.01) [18]. Gender-based differences in neural engagement have also been identified. Female participants exhibited 25% stronger Gamma responses to aesthetic content such as Instagram influencer posts (p<0.01), while male participants showed Beta wave dominance during competitive interactions on platforms like Twitter [31]. These patterns underscore the importance of developing platform-specific interventions tailored to user demographics.

Regulatory actions have begun addressing these neurophysiological concerns. The European Union’s Digital Services Act (2023) introduced "neurowellbeing" requirements, citing EEG evidence of fatigue associated with Delta and Theta wave activity [32]. Likewise, the U.S. Surgeon General’s 2023 advisory warned of developmental risks linked to social media use and recommended design changes like disabling autoplay to reduce Beta and Gamma overstimulation [20]. In response to such concerns, Meta launched its "Take a Break" feature in 2022, pausing app use after 10 minutes. This initiative was based on internal EEG findings showing a statistically significant drop in Alpha power below baseline (p<0.05) [21]. However, critics argue that these measures are largely superficial. Our findings support the need for more robust interventions, such as mandatory app timers informed by real-time neural recovery metrics.

Critical gaps and contributions

While most existing studies have primarily focused on EEG fluctuations during periods of active social media use [13], our research broadens this perspective by investigating neural activity following disengagement. We found that Beta and Gamma activity frequently persists after use, with debates or news-related content delaying Alpha wave recovery by around 15 minutes (p<0.001). Recent EEG research further supports these observations, demonstrating that short-form video consumption on TikTok and Instagram Reels is linked to prolonged Beta and Gamma activity even after exposure ends, contributing to difficulties in attentional refocusing and increased cognitive load [16,22]. Furthermore, unlike previous work that rarely accounted for content-specific differences, our findings emphasize the impact of content type on neural dynamics. Political and news content was shown to suppress Alpha activity 40% longer than lighter content, such as memes (p<0.01) [11]. Additionally, earlier wearable EEG studies have predominantly concentrated on single-platform usage, whereas our results highlight the cognitive burden of multitasking across multiple platforms. Parietal lobe Beta and Theta wave analysis illustrates the increased mental strain from platform switching, informing potential strategies for effective "digital detox" interventions [20]. Taken together, these insights underscore the need to systematically characterize how real-time social media use affects brainwave activity across different content types and user interactions. Therefore, the present study aims to fill this gap by combining high-resolution EEG monitoring with content-specific analysis to delineate both immediate and lingering neurocognitive effects of social media engagement.

## Materials and methods

Brain activity measurement

We used EEG for data monitoring, and collection was done at 512 Hz using a 32-bit EEG recording system with 24 channels, which followed a 10-20 system scalp montage. The 10-20 system ensures consistent electrode placement across different individuals. The system divides the skull into increments of 10% to 20% of the total head size, ensuring that electrodes are placed over all major brain regions.

Participant demographics and selection criteria

We included 100 healthy adults aged 18-45 years (mean age: 27.4 years). The cohort comprised 52 females and 48 males. All subjects denied a history of neurological or psychiatric illness and were examined to rule out the current intake of any drugs that influence the activity of the central nervous system. The socioeconomic backgrounds varied as the participants were recruited in urban and suburban areas via online advertisements posted at the community level.

Experimental procedure and content selection

Participants engaged in 30-minute sessions of social media use. The content was also programmed to reflect a balance of content types: emotionally neutral content (e.g., factual news headlines), emotionally charged content (e.g., political debates and news about crises), visually stimulating content (e.g., high-contrast travel and lifestyle images), and short-form video content (e.g., TikTok clips and Instagram Reels). The engagement metrics and the popularity of the platforms were used to provide content selection, and all participants were exposed to the same content as a way of standardizing the exposure.

EEG data preprocessing

The EEG data underwent a multiple-preprocessing pipeline to ensure the removal of artifacts and to prepare the signal for subsequent analysis. The preprocessing was performed using the MNE-Python toolbox, ensuring high fidelity of brain activity by eliminating various forms of noise and signal distortions.

Band-Pass and Notch Filtering

EEG data is inherently noisy due to various external and physiological sources of interference. To address this, we applied a zero-phase band-pass filter between 0.1 Hz and 50 Hz. Frequencies below 0.1 Hz, usually caused by electrode movements and sweat, were not included, nor were frequencies above 50 Hz, which are mostly due to muscle activity and noise from the surroundings. In addition, a notch filter was used at 50 Hz to eliminate the common power line noise from the data. Thanks to band-pass and notch filtering, we were able to keep brain activity signals and remove unwanted noise from other sources.

Artifact Removal Using Independent Component Analysis (ICA)

To eliminate artifacts arising from eye blinks, muscle movements, and cardiac activity, we applied ICA, a technique that decomposes EEG data into statistically independent components. It helps to eliminate non-neural signals and keep the neural activity intact. Eye blink artifacts were found to have a unique pattern on the frontal areas and moved slowly, mainly impacting the frontal electrodes. Muscle activity was seen as high-frequency oscillations in the scalp close to the facial muscles, and it was mostly found at certain electrodes. The regular and rhythmic patterns of cardiac artifacts in ECG signals were used to identify them. These parts were taken out to avoid cardiovascular noise from affecting the EEG signal. Using ICA, a careful visual review was done to remove only the artifacts from the data, so the real brain signals were not affected.

Re-referencing to Common Average Reference (CAR)

The data were re-referenced to the common average reference (CAR), which is a common way to reduce local reference effects and make signals from different channels more comparable. Here, the average of all electrode signals was taken and then subtracted from each channel’s po- potential. Doing this helps to standardize the EEG signal and decreases local noise, which improves the stability of the following analysis.

Epoching and Baseline Correction

The EEG signal was divided into short sections around each stimulus, so the brain’s response to the experiment could be studied at the same time as the stimuli. Epoching meant taking sections of the EEG data, starting 200 ms before and ending 1000 ms after the onset of every event. To reduce the impact of any noise or drift before the stimulus, a baseline correction was used. Every epoch was adjusted by subtracting the baseline point, which is the average signal strength in the 200-ms period before the stimulus. Thanks to this process, any activity that happened before the stimulus was removed, which helped researchers understand ERPs and other brain changes linked to events.

Downsampling

The EEG data were first recorded at 512 Hz, which gives a clear picture of brain activity but makes processing the data more demanding. The data were reduced to 250 Hz to ensure that the information needed was not lost and the computations could be done more efficiently. The data were reduced, but it still provided enough detail to observe important brain activity in the Delta, Theta, Alpha, Beta, and Gamma bands. This rate is in line with what is used in EEG studies and helps retain all the needed information for time-frequency and event-related analysis.

## Results

**Figure 1:**
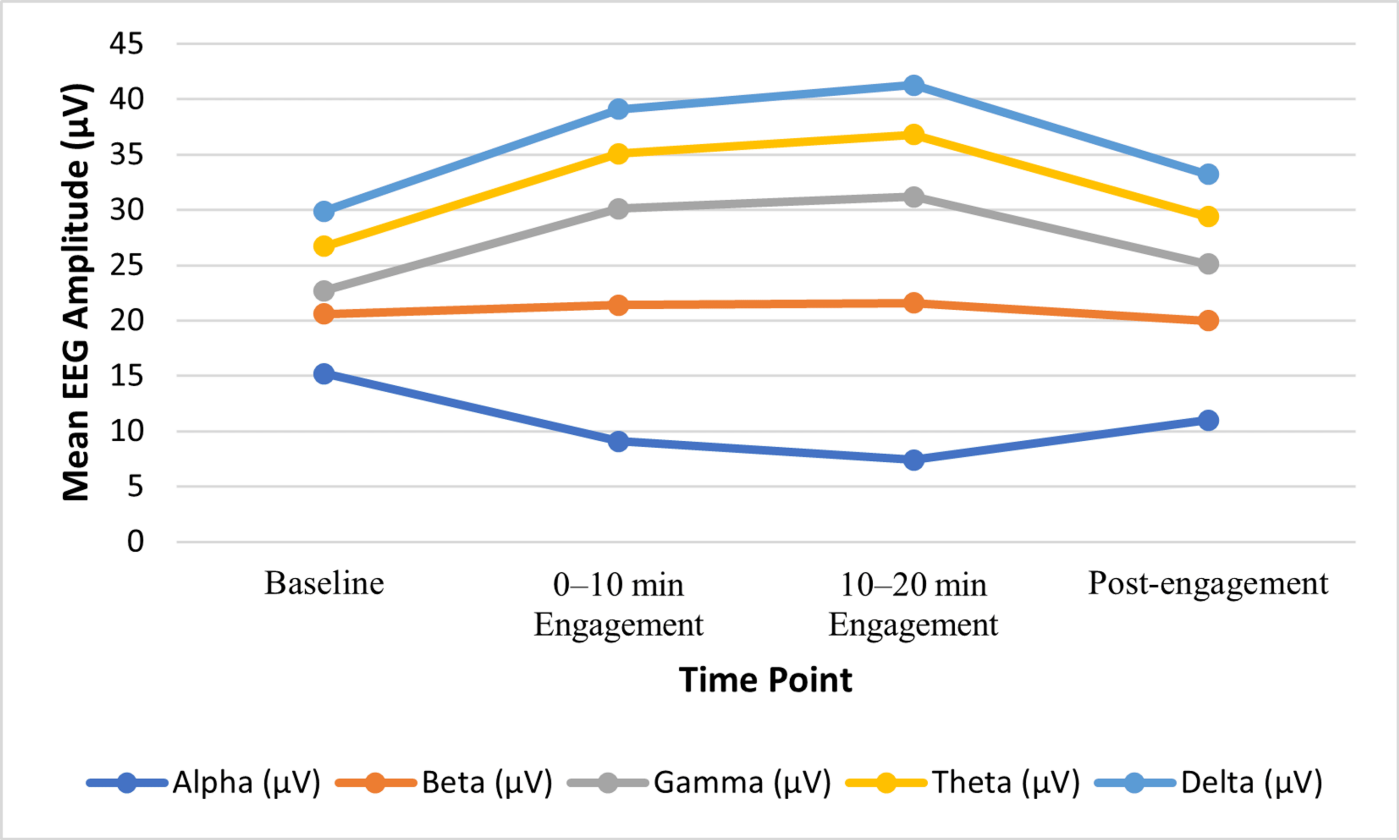
Timeline of EEG band activity during social media use EEG: electroencephalography

During a 30-minute social media session, EEG recordings from 100 participants revealed significant variations in neural activity across the five key frequency bands: Alpha, Beta, Theta, Delta, and Gamma. Figure 1 shows that Alpha amplitude decreased during social media engagement, while Beta and Gamma amplitudes increased and remained elevated after use.

Alpha activity was notably elevated during the baseline period, indicating a relaxed and disengaged mental state. Upon entering social media platforms, Alpha amplitude decreased, reflecting increased cognitive engagement. This suppression was especially prolonged in participants exposed to emotionally or cognitively demanding content, suggesting delayed recovery of a calm neural state post-interaction.

**Figure 2:**
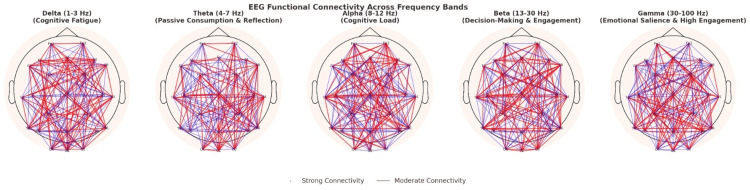
Various wavelengths observed during EEG, their connectivity, and Implications EEG: electroencephalography

In contrast, Beta activity increased substantially during active engagement such as scrolling, commenting, or reacting to content, indicating heightened attention and mental stimulation. This elevation frequently persisted beyond the engagement period, which may suggest continued cognitive excitation or rumination. Gamma activity followed a similar trend, starting low during rest, peaking sharply during exposure to emotionally charged or intellectually demanding posts, and remaining transiently elevated post-interaction. These Gamma waves may reflect higher-order cognitive integration processes triggered by intense media content. Figure 2 illustrates the distinct brainwave patterns and their functional implications during social media use.

Repeated-measures ANOVA revealed significant differences in Alpha amplitude across time points (F(2,198)=24.5, p<0.001). Post hoc comparisons with Bonferroni correction confirmed that Alpha during engagement was significantly lower than baseline (p<0.001) and post-engagement (p=0.02). Beta and Gamma amplitudes also showed significant increases compared to baseline (both p<0.001). Theta and Delta elevations were smaller but reached significance in the final 10 minutes of engagement (p<0.05).

**Figure 3:**
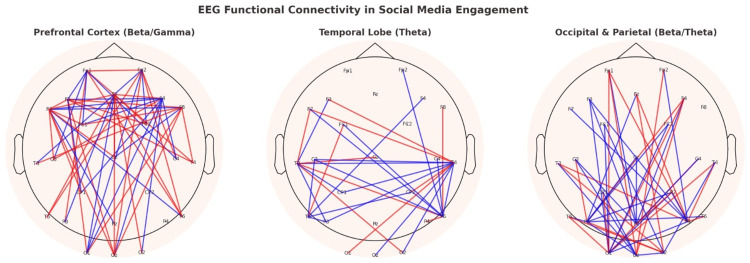
Connectivity of brain regions and lobes, and wavelengths observed as well, and their connectivity EEG: electroencephalography

Figure 3 highlights the regional brain activation patterns associated with social media use, showing Beta and Gamma dominance in the prefrontal cortex during decision-making and emotional evaluation. Theta activity in this region, although less pronounced, likely corresponds to reflective or memory-related processing.

Theta waves overall remained low during rest but rose slightly during passive scrolling or emotionally evocative content. These elevations tended to persist post-engagement, suggesting introspective or self-referential mental states. Delta wave increases were minimal but notable in participants using platforms for extended periods (>20 minutes), likely reflecting mental fatigue. This Delta elevation also lingered in some users' post-session, aligning with cognitive exhaustion patterns.

**Figure 4:**
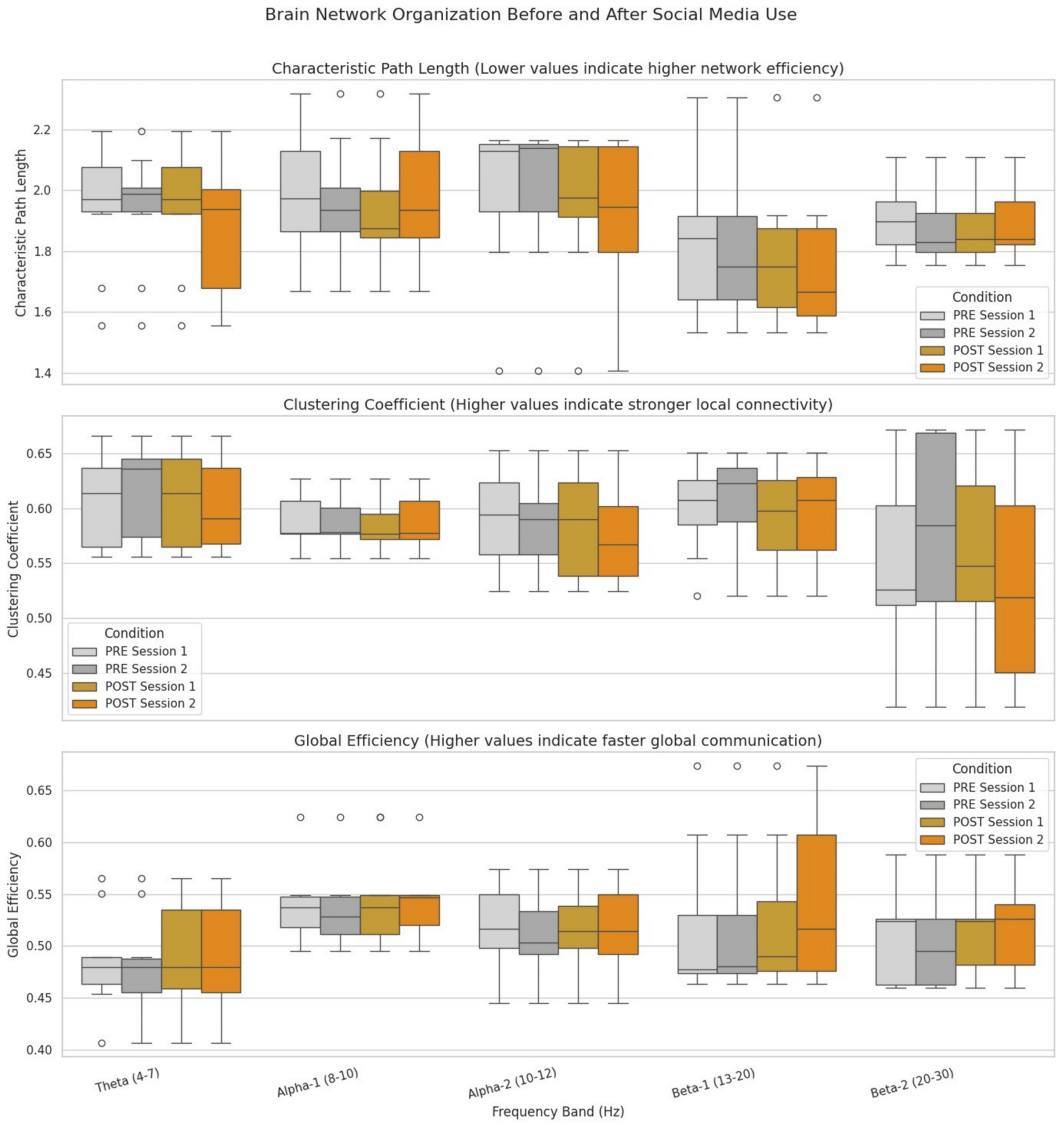
Brain network organization before and after social media use across different frequency bands Frequency bands: Theta = 4–7 Hz; Alpha-1 = 8–10 Hz; Alpha-2 = 10–12 Hz; Beta-1 = 13–20 Hz; Beta-2 = 20–30 Hz. PRE/POST sessions 1 and 2 refer to the first and second sessions before or after the intervention, respectively PRE: pre-intervention; POST: post-intervention

Figure 4 presents pre- and post-use brain network connectivity. It demonstrates an increase in Beta and Gamma connectivity following engagement, accompanied by reduced Alpha coherence. These shifts indicate altered cognitive and emotional states, supporting the conclusion that social media use has a measurable and lasting impact on brain function.

## Discussion

Even though social media has significantly transformed the way people communicate and interact, its underlying influence on neural functioning remains inadequately understood. To explore this, EEG recordings were used to examine the brainwave activity of 100 individuals as they engaged with social media platforms. The results demonstrate that social media use activates multiple frequency bands and brain regions depending on the type of content and user interaction, offering important insights into how digital environments shape cognition and emotion [33,34]. At baseline, Alpha wave activity was elevated, indicating a calm or resting state. Upon exposure to social media content, Alpha activity significantly declined, which is often interpreted as increased cognitive engagement and attentional allocation [35].

Notably, participants who viewed emotionally intense or cognitively demanding content exhibited a slower return to baseline Alpha levels. This suggests that certain types of content may prolong cognitive strain or overstimulation in frequent users [36]. Beta activity increased substantially during active engagement with the platform, such as scrolling, reacting, or navigating. This aligns with Beta's established role in focused attention and goal-directed behavior. Interestingly, Beta activity remained elevated even after users disengaged from the platform, which could imply sustained cognitive processing or rumination. Prior research has similarly linked persistent Beta activity to emotional or personally meaningful thoughts that continue after stimuli are removed [37,38].

Theta waves showed modest fluctuations compared to other bands. Slight increases in Theta were observed during passive content consumption and emotional engagement, consistent with Theta’s role in internal attention and affective processing. Theta activity is also closely associated with the brain’s default mode network (DMN), which is activated during self-referential thinking and daydreaming [39]. This suggests that even passive use of social media may induce introspective states and stimulate the DMN. Longer exposure sessions were associated with mild increases in Delta wave activity, which is typically linked to deep rest and cognitive fatigue.

Users exposed to prolonged content without breaks may not consciously detect fatigue, but EEG data indicate accumulating neural exhaustion. This supports prior findings that link Delta elevations to mental overload and reduced cognitive efficiency in overstimulating environments [40]. Gamma activity rose during the viewing of emotionally intense or mentally challenging posts. Remarkably, Gamma levels remained elevated after the user stopped engaging with the content. This persistence may reflect the brain's continued emotional processing or integration of complex experiences. The observed Gamma response is in line with previous findings on how emotionally charged content can induce prolonged cognitive and emotional effects [41].

Regionally, distinct activation patterns were observed. Increased Beta and Gamma activity in the prefrontal cortex during decision-making and emotional appraisal suggests that reward-related brain networks are engaged during social media use. This parallels findings in studies showing that receiving social rewards (e.g., likes or comments) activates similar circuits as gambling and other reward-seeking behaviors [42]. In the temporal lobe, Theta activity increased during reflective moments, potentially related to autobiographical memory retrieval. The occipital lobe showed more Beta and Gamma activity when participants processed visually rich content, while the parietal lobe exhibited Beta and Theta increases during multitasking or app switching, reflecting higher cognitive load. These findings demonstrate that social media use is a cognitively and emotionally complex activity involving multiple brain regions and oscillatory patterns.

The sustained elevation of Beta and Gamma activity observed in this study may have important implications for mental health, particularly concerning the potential for addictive patterns of social media use. The similarity between reward-related brain activation during social media engagement and the neural mechanisms observed in gambling and substance dependence suggests that digital platforms can reinforce compulsive checking and prolonged engagement behaviors. In addition, the delayed recovery of Alpha rhythms and increases in Delta power after extended use point to accumulating cognitive fatigue, which may contribute to symptoms of digital burnout, reduced attentional capacity, and mood dysregulation over time. These findings underscore the importance of recognizing social media overuse not only as a behavioral concern but also as a neurophysiological phenomenon that could exacerbate existing vulnerabilities in individuals prone to anxiety, depression, or addictive tendencies.

Despite these important insights, several limitations must be acknowledged. Firstly, the cross-sectional design restricts the ability to infer causality or assess long-term changes in brain activity. Second, while EEG provides excellent temporal resolution, its limited spatial resolution constrains precise localization of neural sources. Third, the lab-based setting may not fully capture naturalistic user behavior, potentially impacting ecological validity. Additionally, the sample consisted of healthy adults, which may not reflect responses in adolescents or individuals with cognitive or emotional vulnerabilities. Content and platform variability further introduce uncontrolled factors.

The next step that researchers take should involve longitudinal and real-life studies to monitor the evolution of brainwave patterns. EEG in conjunction with other imaging tools, like fMRI, may allow a more comprehensive picture of brain dynamics to be seen. Individual exposure designs may assist in determining the kind of content that most probably will result in overstimulation or tiredness. It is also necessary to discuss whether there are individual factors that predispose an individual to problematic use, e.g., reward sensitivity or impulsivity. Researchers would be able to investigate whether long-term activation of Beta and Gamma is a predictor of compulsive social media behaviors or life disturbance in the daytime. The other question that is important is whether the long-term changes in resting Alpha power occur after frequent exposure to emotionally intense content.

Future research can also examine whether individuals of greater impulsivity are more prone to Beta and Gamma persistence. Moreover, specific treatments such as digital detox programs or neurofeedback training may be experimented with to understand whether they allow normalizing the brain activity and enhancing concentration. To respond to these questions, researchers might use EEG in combination with digital usage logs and ecological momentary assessment (EMA) to relate brain patterns to real-life behavior and fatigue or craving. The combination of EEG and fMRI might also be used to reveal the precise brain structures that are associated with compulsive use. Lastly, Delta and Theta wave patterns can be monitored to find out the initial symptoms of mental fatigue and assist in the creation of healthier online environments. Finally, Delta and Theta wave patterns may serve as promising neural markers for identifying mental fatigue or compulsive media engagement, paving the way for neurofeedback tools or interface designs that support healthier digital use.

## Conclusions

This study addresses a critical gap in the neuroscience of social media by mapping EEG brainwave patterns to distinct engagement states. By validating previously observed phenomena such as Alpha suppression and Beta/Gamma surges and uncovering new patterns, including Delta-related fatigue and Theta-linked reflection, we offer a structured understanding of how social media captures attention and influences mental well-being. Our findings highlight that prolonged engagement with emotionally charged or visually stimulating content may disrupt cognitive recovery and contribute to mental fatigue, mood disturbances, and compulsive use patterns. These insights may contribute to the development of healthier platform features, such as minimizing Gamma-driven content that triggers prolonged cognitive engagement, as well as clinical strategies for users at elevated risk. Platform designers could consider adaptive timers, content warnings, or customized break prompts informed by real-time neural metrics. Clinicians and researchers may also leverage EEG markers identified here to monitor and manage problematic social media use more effectively. As social media continues to evolve, our tools and approaches for understanding its neural effects must evolve in parallel. Future interdisciplinary collaborations between neuroscientists, technologists, and policymakers will be essential to translate these insights into actionable interventions that support healthier digital habits.
